# A Spanish Pillbox App for Elderly Patients Taking Multiple Medications: Randomized Controlled Trial

**DOI:** 10.2196/jmir.3269

**Published:** 2014-04-04

**Authors:** José Joaquín Mira, Isabel Navarro, Federico Botella, Fernando Borrás, Roberto Nuño-Solinís, Domingo Orozco, Fuencisla Iglesias-Alonso, Pastora Pérez-Pérez, Susana Lorenzo, Nuria Toro

**Affiliations:** ^1^Sant Joan-Alicante Health DistrictConsellería SanidadAlicanteSpain; ^2^Health Psychology DepartmentMiguel Hernández UniversityElcheSpain; ^3^Research Network on Health Services in Chronic Diseases (REDISSEC)MadridSpain; ^4^Center of Operations Research University InstituteMiguel Hernández UniversityElcheSpain; ^5^Statistics, Mathematics and Informatics DepartmentMiguel Hernández UniversityElcheSpain; ^6^Basque Institute for Healthcare Innovation (O+berri)BarakaldoSpain; ^7^Department of Family MedicineFaculty of MedicineMiguel Hernández UniversityAlicanteSpain; ^8^Castilla La Mancha Health Service (SESCAM)ToledoSpain; ^9^Andalusian Patient Safety ObservatoryAndalusian Agency for Health Care QualitySevilleSpain; ^10^Hospital Universitario Fundación AlcorcónMadridSpain

**Keywords:** medication, patient nonadherence, mobile apps, patient safety, elderly

## Abstract

**Background:**

Nonadherence and medication errors are common among patients with complex drug regimens. Apps for smartphones and tablets are effective for improving adherence, but they have not been tested in elderly patients with complex chronic conditions and who typically have less experience with this type of technology.

**Objective:**

The objective of this study was to design, implement, and evaluate a medication self-management app (called ALICE) for elderly patients taking multiple medications with the intention of improving adherence and safe medication use.

**Methods:**

A single-blind randomized controlled trial was conducted with a control and an experimental group (N=99) in Spain in 2013. The characteristics of ALICE were specified based on the suggestions of 3 nominal groups with a total of 23 patients and a focus group with 7 professionals. ALICE was designed for Android and iOS to allow for the personalization of prescriptions and medical advice, showing images of each of the medications (the packaging and the medication itself) together with alerts and multiple reminders for each alert. The randomly assigned patients in the control group received oral and written information on the safe use of their medications and the patients in the experimental group used ALICE for 3 months. Pre and post measures included rate of missed doses and medication errors reported by patients, scores from the 4-item Morisky Medication Adherence Scale (MMAS-4), level of independence, self-perceived health status, and biochemical test results. In the experimental group, data were collected on their previous experience with information and communication technologies, their rating of ALICE, and their perception of the level of independence they had achieved. The intergroup intervention effects were calculated by univariate linear models and ANOVA, with the pre to post intervention differences as the dependent variables.

**Results:**

Data were obtained from 99 patients (48 and 51 in the control and experimental groups, respectively). Patients in the experimental group obtained better MMAS-4 scores (*P*<.001) and reported fewer missed doses of medication (*P*=.02). ALICE only helped to significantly reduce medication errors in patients with an initially higher rate of errors (*P*<.001). Patients with no experience with information and communication technologies reported better adherence (*P*<.001), fewer missed doses (*P*<.001), and fewer medication errors (*P*=.02). The mean satisfaction score for ALICE was 8.5 out of 10. In all, 45 of 51 patients (88%) felt that ALICE improved their independence in managing their medications.

**Conclusions:**

The ALICE app improves adherence, helps reduce rates of forgetting and of medication errors, and increases perceived independence in managing medication. Elderly patients with no previous experience with information and communication technologies are capable of effectively using an app designed to help them take their medicine more safely.

**Trial Registration:**

Clinicaltrials.gov NCT02071498; http://clinicaltrials.gov/ct2/show/NCT02071498 (Archived by WebCite at http://www.webcitation.org/6OJjdHVhD).

## Introduction

### Background

 Chronic disorders are more common among elderly people. In Spain, persons older than 65 years have 3 chronic conditions on average [1]. As many as 94% take 5 or more drugs every day [1,2] and more than half are under the care of more than one doctor [3]. These rates are similar those found in other countries [4,5].

### Safe Medication Use Among Elderly Individuals

The complexity of treatment regimens can lead to an accumulation of medications, confusion because medications look similar (especially in the case of generic medications), and a lack of coordination between the different levels of care. Patients’ intrinsic factors (eg, cognitive impairment and false beliefs regarding some drugs) have been also cited among the most common causes of nonadherence [6] and of involuntary errors related to taking medication at home [3] in Spain. Similar patterns have been observed in other countries [7-13].

### Nonadherence

Improving treatment adherence is a priority because nonadherence to long-term treatments has a negative impact on the health of patients and leads to unnecessary expenditures [8,14,15]. The causes of nonadherence are multiple [16], but it is known that as the complexity of medication regimens increases, there is more likely to be unintentional nonadherence, as observed in diabetes patients [17,18]. That is, nonadherence may be intentional when patients chose not to take the medicines as prescribed, but also unintentional when patients experience difficulties taking the medications [19]. Among other reasons, these difficulties are attributable to one-third of elderly patients failing to assimilate the information required to continue prescribed drug regimens correctly at home [20-23].

### Patient Errors

Taking the wrong dose or the wrong medication accounts for 5% of hospital admissions [24]. In Spain, 35.8% of patients with diabetes admitted to having committed 4 or more medication errors in the previous year [12], and among those who had had diabetes for longer, a higher rate of patients reported drug interactions from taking together drugs that should not be mixed [25]. Sakar et al [10] found that as many as 59% of patients with type 2 diabetes made mistakes with the self-administration of medications and 21% with diet.

### The Current Study

The participation of patients in their own care [26,27] has been shown to help improve use of drugs, for example, associating medications with specific meals, writing notes on the packaging, and using pillboxes [28,29]. Recently, with the growth in use of tablets and smartphones, various interventions [30-32] have been designed to improve adherence and it has been found that such tools are effective and help to increase patient independence [33].

Usage of apps for tablets and smartphones (eg, iOS, Android, Windows Phone, and Blackberry OS) designed to improve adherence has increased exponentially [34]. However, these apps have various limitations [35,36]: they are conceived for patients familiar with these technologies, they have not been written for elderly patients (assuming that they would not benefit from this type of tool), and in most cases, patients have not been consulted about the design [37]. Few are available in Spanish or in languages other than English, in general, reducing their use among those who are not English speakers. Furthermore, no studies have tested the effectiveness of these apps with the elderly.

The objective of this study was to design, implement, and evaluate a medication self-management app in Spanish for elderly patients taking multiple medications by using tablets (Android or iOS) to increase adherence to treatment regimens and achieve safer use of drugs.

## Methods

### ALICE Design

A tablet-based medication self-management app (called ALICE) was designed to help patients to remember to take all their medications at the correct doses, distinguish between drugs to avoid confusions, avoid known potential interactions and common errors in use of the medications, and know how to properly store the medications. ALICE was also designed to remember doctors’ recommendations for healthy habits, such as physical exercise and diet. The app design was based on suggestions extracted from 3 nominal groups with a total of 23 patients and a focus group with 7 professionals (3 physicians and 4 pharmacists). The guide with questions for patients and professionals was drawn up considering the classification of medication errors in the study by Field et al [13] at the Meyers Primary Care Institute in Massachusetts and previous research by our group on patients’ perception of safety and the frequency and characteristics of patients’ medication errors [3].

### Tablets Used

The tablet used was selected on the basis of the need for a device with an at least 7-inch easy-to-use touch screen, ensuring that users would only have to follow simple instructions and tap on some icons on the screen. Specifically, the BQ Verne Plus 3G with an liquid-crystal display (LCD) tactile screen was chosen for an Android and an iPad 2 with Wi-Fi and 3G for the iOS.

### ALICE Characteristics

**Figure 1:**
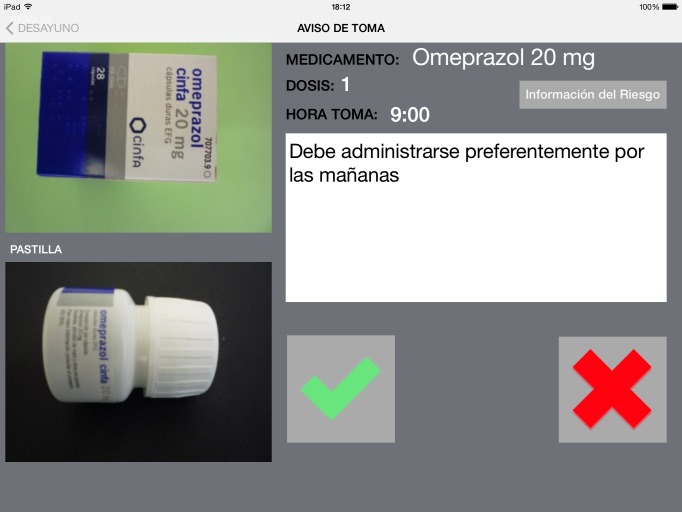
Example of a ALICE screen.

The ALICE app was designed to work with personalized prescriptions and recommendations given to patients, with a function making it possible to store details of all their prescriptions and related instructions, as well as images for each of the medications (even allowing pictures to be taken of the packaging or the appearance of the medication itself), and recommendations of the various different doctors seeing the same patient. A second function established a customized system of alerts and reminders for a given alert, to remind patients when to take their medications and to put into practice healthy habits, using the approaches suggested by participants in the nominal groups (eg, the association of the intake of medicines with particular meals or daily activities, and various techniques for splitting pills). Lastly, a third function was to enable monitoring of the level of adherence to the prescriptions and medical advice, the tablet connecting via a wireless or 3G network with the study monitoring system, with the health care provider and with a relative or caregiver when authorized by the patient (Figure 1).

When it is time to take a medication, an alarm sounds and the patient accesses the main menu of the app. Displayed is the name of the medication, dose, time, and any warnings of how the patient should take the medication (if applicable). The patient may consult his/her medication anytime. The medication usually is associated with breakfast, lunch, snack, or dinner daily. ALICE follows this normal use. The ALICE app reports medications the patient must take in a day and reports medicines that the patient has forgotten to take that day.

Once the tool was designed and before the experimental phase, we assessed its feasibility, verifying that all the app characteristics proposed in the previous phase were included in the design process. For this purpose, 8 elderly patients assessed the user-friendliness of the tool, its degree of intuitiveness, whether the font was sufficiently large, the contrast of the text and images, and the quality of the photographs. Identified problems were the low volume of alerts and a proposal to simplify the system for taking photos of drugs to include images on ALICE. Both problems were solved.

The ALICE app has the following features: a user-friendly interface to introduce text and images; various medication reminder alerts and messages for patients, including sounds or flashing and also messages sent to relatives or caregivers; and a complete list for the caregivers of all prescriptions regardless of the number of doctors involved including a summary of patient adherence behavior.

### Study Design

To evaluate ALICE we opted for a single-blind experimental design with 2 groups (control and experimental) and pre and post assessments using a randomized controlled trial (NCT02071498). Patients were randomly assigned to the control or experimental group. The control group was composed of participants who did not use ALICE, and the experimental group was composed of people who used this tool for 3 months. To maintain the blinding and be able to link the pre and post measurements, patients were assigned codes based on their date of birth and initials.

### Patients Enrolled

We randomly selected 102 patients with a digital medical history from 13 health centers in the health districts of Alicante and Bilbao. We defined the following inclusion criteria for the study: multimorbid patients taking multiple medications, older than 65 years, with a Barthel [38] score of more than 60, living in their own home, and able to manage the administration of their medication at home. The sample size was calculated to detect a difference between means of at least 10 points with a statistical power of 90% at a level of significance of alpha=.05 (in a 2-tailed test). We requested the informed consent of patients from both control and experimental groups. This study was approved and financed by the Spanish Ministry of Health, Equality, and Social Policy. The Spanish Research Health Agency (FIS), Independent Clinical Research, project number EC11-527. The Experimental Research Ethics Committee of Miguel Hernández University (DPS-JJM-003-11) approved the trial.

### Measures

All participating patients completed a questionnaire to assess the rates of missed doses and of medication errors, and adherence to treatment measured by the 4-item *Morisky Medication Adherence Scale (MMAS-4)*, as well as self-perceived health status, the number of doctors seen, whether they used a physical pillbox, and who organized their medication (over the previous 3 months). Additionally, we recorded data on the sociodemographic characteristics of participants, namely sex, age, and civil status. Those in the control group received oral and written information regarding the main risks related to their medications and the most common errors of patients when taking medications. Participants in the experimental group were given a BQ tablet or an iPad with the ALICE app installed and personalized according to the medications they had been prescribed as listed in their medical record. Patients in this group attended individual sessions of up to 2 hours to be shown how to use the app. During the study period, patients from the experimental group had a contact telephone number for any query regarding the use of the tablet or the app.

Three months later, the measurements made preintervention were repeated and extra information was collected regarding treatment adherence based on the data provided by ALICE (post). Additionally, patients from the experimental group answered a series of questions to ascertain whether they, prior to the study, had experience with using tablets, smartphones, mobile phones, and computers. They were also asked to evaluate ALICE (its performance, functionality, usability, reliability, acceptability, usefulness, design, simplicity, accessibility, and problem-solving power, as well as overall satisfaction with the tool), whether they would recommend the app to relatives, friends, and acquaintances, and their perception of the degree of independence they had achieved because of ALICE.

### Statistical Analysis

To assess the effectiveness of ALICE, we built various univariate linear models. Where there were intergroup differences in preintervention measurements, we performed univariate linear model and ANOVA tests, using as the dependent variables the differences between the pre and post intervention measurements in MMAS-4 scores, self-perceived health status, levels of glycated hemoglobin (HbA1), low-density lipoprotein (LDL) cholesterol, blood pressure, number of medication errors, and missed doses related to medication reported by patients. Allocation to the experimental or control group was considered as the independent variable. In the experimental group, we carried out Pearson correlation analysis between MMAS-4 scores and treatment adherence assessed objectively by the number of alerts that had not been acknowledged or had been acknowledged late (data provided by ALICE). Additionally, to allow for the potential effect of the level of experience of patients with information and communication technology (ICT), we compared the pre and post intervention measurements in the experimental group adjusting for the effect of familiarity with ICTs. We compared the rating of ALICE by patients in the experimental group with and without ICT experience by using the chi-square test and their level of satisfaction with ALICE (on a scale of 0 to 10) by using the Student *t* test. In all cases, we checked that the assumptions of the statistical tests used were met. We considered *P*<.05 to be statistically significant.

## Results

### Qualitative Study: ALICE Characteristics

According to the 23 patients and 7 health professionals that participated in the qualitative research process, the design of the ALICE app had to satisfy the following criteria: it needed be easy to use, have sufficiently large font size, include photographs of the medications, and offer a large variety of alerts to be set to suit the preferences of patients with a menu enabling patients to easily adjust the alerts to be associated with their breakfast, lunch, dinner, and other meals and snacks, or other daily activities occurring at regular times. For each alert, it had to be possible to program up to 5 reminders and include information to avoid confusion between medicines, and advice on how to take the medication and how to store it properly. It was important to include all the medications patients were on regardless of the prescribing doctor and include automatic reminders of other medical recommendations (eg, to do physical exercise, including what type and for how long). Further, ALICE had to record the level of treatment adherence, send short message service (SMS) text messages to relatives or caregivers if patients failed to adhere to treatments, and alert a monitoring system in the event of a malfunction. Lastly, ALICE had to provide information about all the medications taken by patients, regardless of the number of prescribing doctors, and send reports on medication alerts that were not acknowledged so that when patients attended appointments, their doctors would know the extent to which they were adhering to treatments prescribed.

### Experimental Study: Participant Characteristics

**Figure 2:**
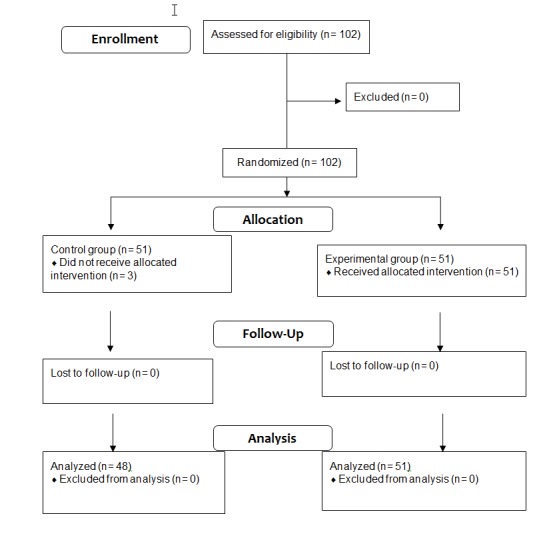
Study flow diagram.

This study was carried out between June 2012 and May 2013. Data were obtained from 99 patients (48 and 51 in the control and experimental groups, respectively). Three patients in the control group declined to participate (Figure 2). The characteristics of those who participated are summarized in Table 1. A total of 72 of 99 patients (73%) took more than 5 types of drugs per day and 36 of 99 (36%) were under the care of more than one doctor. In the experimental group, 22 of 51 (43%) had a computer and 19 of 51 (37%) had an Internet connection at home, whereas 39 of 51 (76%) had a mobile phone and 9 of 51 (18%) a smartphone. On the other hand, 28 of 51 individuals (55%) were not familiar with ICTs, having never used a computer, tablet, or smartphone.

**Table 1:** Description of patients in the experimental and control groups (N=99).

Demographic characteristics		Control n=48	Experimental n=51	*P* ^a^
Age (years), mean (SD)		72.9 (6)	70.9 (8)	.16
**Sex, n (%)**				
	Women	23 (48)	21 (41)	.50
	Men	25 (52)	30 (59)	
**Civil status, n (%)**				
	Single	1 (2)	1 (2)	.10
	Married	39 (81)	32 (63)	
	Widowed	8 (17)	14 (27)	
	Divorced	0 (0)	4 (8)	
**Living arrangements, n (%)**				
	Alone	9 (19)	12 (23)	.56
	With partner/relative	39 (81)	39 (76)	
**Who organized their medication, n (%)**				
	Patient himself/herself	41 (85)	47 (92)	.29
	Partner/relative/caregiver	7 (15)	4 (8)	
**Self-perceived health status, n (%)**				
	Poor	5 (10)	2 (4)	.64
	Fair	14 (29)	17 (33)	
	Good	26 (54)	28 (55)	
	Excellent	3 (6)	4 (8)	
**Disorders** ^b^				
	Diabetes	46 (96)	43 (84)	.06
	Insulin-dependent patients^c^	9 (20)	15 (36)	.09
	Depression/anxiety	5 (10)	4 (8)	.66
	Hypercholesterolemia	24 (50)	28 (55)	.62
	Benign prostatic hyperplasia^d^	3 (75)	7 (14)	.22
	High blood pressure	40 (83)	38 (74)	.28
	Other cardiovascular diseases	22 (46)	21 (41)	.64
	Arthrosis	11 (23)	9 (18)	.51
	Renal failure	8 (89)	6 (12)	.48
	Chronic obstructive pulmonary disease	10 (21)	9 (18)	.69
	Digestive disorders	11 (23)	4 (8)	.04
Number of drugs prescribed, mean (SD)		7.9 (3)	7.6 (3)	.55
Number of doctors involved, mean (SD)		2.3 (1)	2.3 (2)	.99

^a^Based on the Pearson chi-square test or the Student *t* test for independent samples.

^b^Patients could have more than 1 disorder.

^c^Percentage calculated over the total number of patients with diabetes.

^d^Percentage calculated over the total of men in the sample.

### Effects of ALICE

Patients from the experimental group reported greater treatment adherence (measured using the MMAS-4) and a lower rate of missed doses at the end of the study (Table 2). Specifically, treatment adherence (measured by the scale scores) increased by 28.3% and the rate of missed doses fell by 27.3%. On the other hand, the univariate linear analysis of pre-post differences in the control and experimental groups indicated that ALICE was not effective in reducing the rate of medication errors. For this variable, we conducted ANOVA to control for the effect of the difference in the rate of medication errors between the experimental and control groups before the intervention. This analysis indicated that ALICE did help to reduce the medication errors, but only in patients who had recognized that they had made 2 or more errors before the study (Table 2). Among these patients, the rate of medication errors fell by 41.2% compared to before the study.

The patients in the control and experimental groups had similar levels of glycated hemoglobin and blood pressure and similar self-perceived health status before and after the study. However, the pre-post measurements of cholesterol were statistically different; levels increased by 5% compared to before the study (Table 2).

A total of 31,383 medication alerts were programmed. Between Monday and Sunday, 3293 (10.49%) alerts were not been dealt with and 2978 (9.49%) were dealt with after the first reminder. The Pearson correlation coefficient between the number of alerts that were not dealt with and the MMAS-4 score was .3 (*P*=.04). This result showed the positive and significant relationship between the adherence of the MMAS-4 and the information provided by ALICE on the level of patient compliance.

**Table 2:** The 4-item Morisky Medication Adherence Scale (MMAS-4) scores and rates of missed doses and medication errors reported by patients in the control and experimental groups.

Measures		Control group (n=48)		Experimental group (n=51)		Pre-post difference		Effect size Δ	Between-group difference, *P*	
		Pre	Post	Pre	Post	Control	Experimental		ULM^a^	ANOVA
MMAS-4, mean (SD)		7.2 (0.9)	7.3 (0.7)	6.6 (1.2)	7.4 (0.9)	0.1	0.8	0.7	<.001	—
Self-perceived health status, n (%)		68.3 (21)	69.1 (20)	71.27 (17)	74.6 (17)	0.9	3.3	1.2	.54	—
Glycated hemoglobin (mmol/mol), mean (SD)		7.1 (1.1)	6.7 (1.4)	7.1 (1.4)	7.4 (2.7)	–0.4	0.3	0.4	.36	—
Cholesterol (mg/dL), mean (SD)		105.4 (29.9)	101.9 (28.1)	107.0 (29.8)	112.7 (45.8)	–3.5	5.7	12.2	.04	—
**Blood pressure (mm Hg), mean (SD)**										
	Diastolic	75.8 (10.5)	76.6 (11.2)	72.3 (10)	70.6 (8.8)	0.8	1.7	0.2	.89	—
	Systolic	137.3 (12.4)	140.5 (14.6)	130.9 (15)	128.6 (20.9)	3.2	2.3	2.6	.28	—
**Medication errors,** ^b^ **n (%)**						0.0	–0.1	0.2	.21	—
	0	42 (87)	43 (90)	38 (74)	43 (84)					
	1	6 (12)	3 (6)	9 (18)	6 (12)					.95^c^
	2	0 (0)	2 (4)	4 (8)	2 (4)					<.001
**Number of missed doses, n (%)**						0.2	–0.3	0.5	.02	—
	0	28 (58)	20 (42)	18 (35)	27 (53)					
	1	12 (25)	17 (35)	21 (41)	16 (31)					
	2	6 (12)	9 (19)	8 (16)	5 (10)					
	≥3	0 (0)	2 (4)	4 (8)	3 (6)					

^a^ULM: univariate linear model.

^b^Wrong drug taken (attributed to confusion between drugs that appear similar) or incorrect doses.

^c^For the subgroup of 0 or 1 medication errors reported at the first assessment.

### Experience With Information and Communication Technology

Patients from the experimental group who were not familiar with ICTs obtained higher MMAS-4 scores and reported fewer medication errors and missed doses after the intervention (Table 3). The pattern was similar in patients from the experimental group who had experience with ICTs, except that there was not a significant reduction in the number of missed doses.

**Table 3:** Influence of ALICE and patients’ previous experience with information and communication technology (ICT) in the experimental group (n=51).

Measures		Some ICT experience (n=23)			No ICT experience (n=28)		
		Pre	Post	*P* ^a^	Pre	Post	*P* ^a^
Morisky Medication Adherence Scale, mean (SD)		6.6 (1.4)	7.4 (0.8)	.01	6.6 (1.1)	7.2 (1.0)	<.001
**Medication errors,** ^b^ **n (%)**							
	0	18 (78)	19 (83)		20 (71)	24 (86)	
	1	3 (13)	3 (13)		6 (21)	3 (11)	
	2	2 (9)	1 (4)	.01	2 (7)	1 (4)	.02
**Missed doses reported by patients, n (%)**							
	0	9 (39)	14 (61)		9 (32)	13 (46)	
	1	9 (39)	6 (26)		12 (43)	10 (36)	
	2	4 (17)	2 (9)	.14	4 (14)	3 (11)	<.001
	≥3	1 (4)	1 (4)		3 (11)	3 (7)	

^a^Based on the Wilcoxon test for paired samples (MMAS-4) or differences based on phi and Cramer’s V (medication errors and missed doses).

^b^Wrong drug taken (attributed to confusion between drugs that appear similar) or incorrect errors.

### ALICE Functioning

More than half of the patients from the experimental group (30/51, 59%) required individual support once they joined the study to solve problems related to the use of ALICE. Most of these (9/29, 31%) concerned charging the battery and restarting the system (Table 4).

**Table 4:** Patient assessment of the ALICE functioning.

Patient report		n (%)
**Has the tablet worked?**		
	Yes, it has consistently worked well from the beginning	21 (41)
	Yes, though I have had some problems	29 (57)
	No, it has consistently failed to work	1 (2)
**In the event that it has failed to work, what was the problem?**		
	It kept turning off	6 (21)
	The alert didn’t trigger	10 (34)
	The sound was poor	1 (3)
	The battery ran down very quickly	3 (10)
	It froze and/or crashed	5 (17)
	Other	4 (14)
**Was the problem solved quickly and effectively?**		
	No, the problem was not solved	1 (3)
	Yes, the problem was always solved	28 (96)

### Participant Satisfaction and Medication Self-Management

The level of satisfaction with ALICE (in the experimental group) was similar in those with some and with no previous ICT experience (Table 5). The mean scores were more than 8 out of a maximum of 10. More than half of the patients in the experimental group (30/51, 59%) reported that the ALICE app improved their medication use. Another 15 (29.4%) considered that ALICE helped to a certain extent, whereas 6 patients (11.8%) indicated that ALICE did not help at all (Table 5).

**Table 5:** Patient satisfaction with ALICE app.

ALICE satisfaction		Some ICT experience n=23	No ICT experience n=28	Total N=51	*P* ^a^
**Patient report, n (%)**					
	I like the design of the messages and alerts in ALICE	23 (100)	28 (100)	51 (100)	—
	The photos of the pills/capsule help me take the correct drug	23 (100)	28 (100)	51 (100)	—
	The font size is sufficiently large	23 (100)	27 (96)	50 (98)	.36
	The photos of the medication packaging help me take my medication correctly	23 (100)	27 (96)	50 (98)	.36
	The instructions for using ALICE have been clear, correct and complete	22 (96)	27 (100)	49 (98)	.27
	ALICE is easy to use and manage	21 (91)	27 (96)	48 (94)	.53
	It is sufficiently large to see the screen well	18 (78)	27 (96)	45 (88)	.13
	It is easy to tap on the correct icon with my finger	21 (91)	24 (86)	45 (88)	.28
	In general, it is easy to operate with my finger	20 (87)	24 (86)	44 (86)	.46
	The audio alerts are loud enough	20 (87)	22 (79)	42 (82)	.27
Overall satisfaction with ALICE, mean (SD)		8.3 (1.4)	8.7 (1.4)	8.5 (1.4)	.26

^a^Based on the chi-square test *t* test for unpaired samples (overall satisfaction).

## Discussion

### Principal Results

Treatment adherence was higher in patients in the experimental group than in the control group. It is important to highlight that the ALICE app helped to solve a common problem in elderly patients; namely, remembering whether they have taken their medication. By using ALICE, the number of errors decreased, although the patients who benefited in this respect were those who previously made the most errors. Our data suggest that ALICE does contribute to reducing systematic errors, but not all medication errors.

The self-perceived health status, levels of glycated hemoglobin, and blood pressure remained similar from the beginning to the end of the study, and although cholesterol levels increased, the changes were not clinically relevant. That is, ALICE did not improve the clinical status of patients; however, 3 months may not be long enough to observe differences. It did, however, improve patients’ perception of independence in the management of their medications.

Elderly patients with complex drug regimens, even those without previous experience in the use of tablets, smartphones, and computers, or who had never used the Internet, were found to be capable of effectively using an app designed to improve safe medication use. ALICE was designed to cope with attrition of users using these devices. ALICE was used daily and only 1 patient left the study because of a problem with the tablet and not with ALICE.

### Comparison With Previous Studies

This is one of the few experimental studies assessing the effectiveness of a virtual pillbox for tablets and smartphones targeted to elderly patients with multiple health problems. The design of ALICE took into account the findings of other studies, but was based on the views and preferences of the target patients and the experience of the primary care and pharmacy personnel caring for them. The suggestion to assess the usability during the development of this intervention was also applied [39]. This approach to designing an app has not been widely implemented, but it has been proposed in other studies focused on developing such tools. Indeed, this development process gave rise to one of its strengths, the fact that it can be adapted to the habits, timetables, and lifestyles of each patient as suggested by Tatara et al [37]. In adherence studies, the personalization of tools has not been widely considered and our results underline the importance of the participation of patients and professionals in the design of applications.

Traditional pillboxes improve treatment adherence, but are only useful in the case of solid dosage medications [40]. Further, although they help with the organization of medications at home, elderly patients may find it difficult to put their pills into the compartments [41]. One study found that approximately 22% of patients were unable to effectively use physical pillboxes [42]. It might be possible to overcome these limitations by using a virtual pillbox for tablets and smartphones.

The level of adherence, assessed in 3 different ways (direct reporting, MMAS-4 scores, and statistics from ALICE on unacknowledged alerts), was good in all cases compared to the mean nonadherence figures reported for chronic patients in general [19] (approximately 50%), being slightly higher in patients with diabetes, which was one of the most common diseases in our study population, and was slightly lower than in another study of a pillbox for smartphones that obtained adherence of 36%. Similar to other studies, ALICE showed capacity to modify the behavior of adult patients increasing adherence to the therapeutic regimen and reducing medication errors in some cases [43].

### Relevance of This Study

ALICE-like apps make it possible to provide information that contributes to safer use of drugs by tuning in to the needs of specific patients, for example, by including advice and preventive measures that the doctors involved consider the most appropriate (given the medication dose or timings). Further, they can be adapted to the habits and lifestyle of patients, to make it easier and simpler for them to take their medication because it is known that these factors are the main cause of nonadherence. These issues have been taken into account in the design of ALICE and it seems this app does have a beneficial effect on the level of adherence and patients perceive that it improves the way they take their medication.

Rates of medication errors are particularly high in patients on multiple medications [11,12,27]. One of the objectives when designing ALICE was to reduce medication errors among elderly patients with multimorbidity by providing a tool that they could use themselves. This objective is particularly important from a clinical point of view in the target population. Field et al [13] found that most medication errors made by patients concerned oral hypoglycemic agents (29%) and more than one-third (36%) of our patients were on this type of drug. However, this objective was only partly achieved.

### Limitations

The small number of participants and the number of months using ALICE affected our ability to detect differences between the group using the ALICE app and the control group (eg, in relation to biomarkers) as well as our ability to generalize the results. This type of app does not address the issue of where to store medication at home to have it accessible when it should be taken. The data collected do not include details about the number of drugs to be taken daily. No count was made of pills nor did we use any other system to check if patients actually took any medication after acknowledging the alert. Further, we cannot be sure that they took the correct dose or even the correct drug. We do not know whether ALICE would continue to be effective in the longer term. This is important because a notable reason patients cite for stopping taking medications is the feeling that they are not working for them. There is some evidence that the *MMAS-4* overestimates adherence, yielding higher rates than those obtained from pill counts. This study did not consider medication reconciliation. In some cases, if there has been inappropriate prescribing, following treatment regimens could pose a risk. This app only addresses unintentional nonadherence. In the event of a patient being reluctant or opposed to taking a certain medicine [44] (this known to affect approximately 43% of cases), this type of tool might be useful for its educational features, but exploring this issue is beyond the scope of this study.

### Recommendations for Practice and Research

Most apps have been designed for patients with less complex health problems and/or experience with ICTs. This study should change the expectations of developers and mobile phone companies, encouraging them to develop apps and devices suited to older patients with multimorbidity who are normally excluded from studies thought to be too complex because such tools could improve the capacity of these individuals to manage their illnesses.

Glucose monitors and other devices currently in use in telemedicine programs could have add-ons to help individual patients use their medications more safely. Such devices should not only include alerts for medicines, but also reminders of how to put advice on healthy habits into practice.

Further studies on virtual pillboxes for tablets and smartphones could explore whether adherence can be improved by personalization of treatment regimens as suggested by other authors [45]. Specifically, future research should assess to what extent these tools are useful for older individuals living alone, a situation that is expected to be the reality for a growing number of patients in the near future.

ALICE and other similar apps have a broad potential not only for patients, but also for professionals because they can provide useful information about how patients adapt the therapeutic regimen to their lifestyle. Future studies could explore how to improve physician recommendations to increase adherence to treatment based on personalized information.

## Multimedia Appendix 1

CONSORT-EHEALTH checklist V1.6.2 [46].

## Abbreviations

ICT: information and communication technology
LCD: liquid-crystal display
LDL: low-density lipoprotein
MMAS-4: 4-item Morisky Medication Adherence Scale
SMS: short message service
