# Supplementary material for: A Spanish Pillbox App for Elderly Patients Taking Multiple Medications: Randomized Controlled Trial
Source: J Med Internet Res. 2014 Apr 4;16(4):e99. doi: 10.2196/jmir.3269 (PMC4004137; doi:10.2196/jmir.3269)
Supplement: Supplementary file 1 [file jmir_v16i4e99_app1.pdf]

CONSORT-EHEALTH Checklist V1.6.2 Report

(based on CONSORT-EHEALTH V1.6), available at [http://tinyurl.com/consort-ehealth-v1-6].

3269

Date completed

2/2/2014 6:01:27

by

MIRA

A Spanish pillbox app for elderly patients taking multiple medications: Randomized controlled trial

TITLE

1a-i) Identify the mode of delivery in the title

Introduction: Safe medication use among elderly individuals, Non-adherence, Patient errors, Tools for patient self-management, The current study
Methods: ALICE design, Tablets used, ALICE characteristics, Study Design, Patients enrolled, Measures, Statistical analysis
Results: Qualitative study. ALICE characteristics, Experimental study. Participant characteristics, Effects of ALICE, Experience with ICTs, ALICE functioning, Participant satisfaction, Patient medication self-management
Discussion: Main results,Comparison with previous studies, Relevance of this study, Limitations, Recommendations for practice and research

1a-ii) Non-web-based components or important co-interventions in title

1a-iii) Primary condition or target group in the title

for elderly patients taking multiple medications

ABSTRACT

1b-i) Key features/functionalities/components of the intervention and comparator in the METHODS section of the ABSTRACT

ALICE was designed for Android and iOS and to allow the personalisation of prescriptions and medical advice, showing images of each of the medications (the packaging and the medication itself) together with alerts and multiple reminders for each alert

1b-ii) Level of human involvement in the METHODS section of the ABSTRACT

The subjects in the control group received oral and written information on the safe use of their medications and the experimental group used ALICE for three months

1b-iii) Open vs. closed, web-based (self-assessment) vs. face-to-face assessments in the METHODS section of the ABSTRACT

The randomly subjects in the control group received oral and written information on the safe use of their medications and the experimental group used ALICE for three months.

1b-iv) RESULTS section in abstract must contain use data

Data were obtained from 99 patients (48 and 51 in the control and experimental groups, respectively).

1b-v) CONCLUSIONS/DISCUSSION in abstract for negative trials

ALICE improves adherence, helps to reduce the rates of forgetting and of medication errors, as well as increasing the perceived independence in managing medication. Elderly with no previous experience with information and communication technologies are capable of effectively using an app designed to help them take their medicine more safely

INTRODUCTION

2a-i) Problem and the type of system/solution

Usage of apps for tablets and smartphones (with iOS, Android, Windows Phone and Blackberry OS, among others) designed to improve adherence has increased exponentially . However, to date, these apps have various limitations , : they are conceived for patients familiar with these technologies, they have not been written for elderly patients (assuming that they would not benefit from this type of tool) and, in most cases, patients have not been consulted about the design while few are available in Spanish and in general in languages other than English, reducing their use among those who are not English speakers. Furthermore, no studies have tested the effectiveness of these apps

2a-ii) Scientific background, rationale: What is known about the (type of) system

Improving treatment adherence is a priority as non-adherence to long-term treatments has a negative impact on the health of patients and leads to unnecessary expenditure.
Sakar et al found that as many as 59% of patients with type 2 diabetes made mistakes with the self-administration of medications and 21% with diet. The participation of patients in their own care, has been shown to help to improve use of drugs, for example, associating medications with specific meals, writing notes on the packaging and using pillbox.

METHODS

3a) CONSORT: Description of trial design (such as parallel, factorial) including allocation ratio

The objective of this study was to design, implement and evaluate a medication-self management app in Spanish for elderly patients taking multiple medications using tablets (Android or iOS) in order to increase adherence to treatment regimens and achieve a safer use of drugs

3b) CONSORT: Important changes to methods after trial commencement (such as eligibility criteria), with reasons

The app design was based on suggestions extracted from 3 nominal groups with a total of 23 patients and a focus group with 7 professionals (3 physicians and 4 pharmacists). The guide with questions to be posed to patients and professionals was drawn up considering the classification of medication errors in the study by Fields at the Meyers Primary Care Institute, Massachusetts13 and previous research by our group on patients' perception of safety and the frequency and characteristics of patients' medication errors3.

3b-i) Bug fixes, Downtimes, Content Changes

ALICE was designed to work with personalised prescriptions and recommendations given to patients, with a function making it possible to store details of all their prescriptions and related instructions, as well as images for each of the medications (even allowing pictures to be taken of the packaging or the appearance of the medication itself), and recommendations of the various different doctors seeing the same patient. A second function established a customized system of alerts and reminders for a given alert, to remind patients when to take their medications and to put into practice healthy habits, using the approaches suggested by participants in the nominal groups (for example, the association of the intake of medicines with particular meals or daily activities, and various techniques for splitting pills). Lastly, a third function was to enable monitoring of the level of adherence to the prescriptions and medical advice, the tablet connecting via a wireless or 3G network with the study monitoring system, with the GP and with relative or caregiver when authorized by the patient.

4a) CONSORT: Eligibility criteria for participants

We randomly selected 102 patients with a digital medical history from 13 health centres in the health districts of Alicante and Bilbao. We defined the following inclusion criteria for the study: multimorbid patients taking multiple medications, over 65 years old, with a Barthel score of more than 60, living in their own home and able to manage the administration of their medication at home. The sample size was calculated to detect a difference between means of at least of 10 points with a statistical power of 90% at a level of significance of $\alpha = 0.05$ (in a two-tailed test). We requested the informed consent of patients from both control and experimental groups. The procedure was approved by the Ethics Committee of Miguel Hernández University (DPS-JJM-003-11).

4a-i) Computer / Internet literacy
ALICE was designed to be used by patients without experience using tablets or computer.
4a-ii) Open vs. closed, web-based vs. face-to-face assessments:
We randomly selected 102 patients with a digital medical history from 13 health centres in the health districts of Alicante and Bilbao. We defined the following inclusion criteria for the study: multimorbid patients taking multiple medications, over 65 years old, with a Barthel score of more than 60, living in their own home and able to manage the administration of their medication at home
4a-iii) Information giving during recruitment
Those in the control group received oral and written information regarding the main risks related to their medications and the most common errors of patients when taking medications. Participants in the experimental group were given a BQ tablet or an iPad with the ALICE app installed and personalised according to the medications they had been prescribed as listed in their medical record. Patients in this group attended individual sessions of up to two hours to be shown how to use the app. During the study period, patients from the experimental group had a contact telephone number for any query regarding the use of the tablet or the app
4b) CONSORT: Settings and locations where the data were collected
Three months later, the measurements made pre-intervention were repeated and extra information was collected regarding treatment adherence based on the data provided by ALICE (post). Additionally, subjects from the experimental group answered a series of questions to ascertain whether they, prior to the study, had experience of using tablets, smartphones, mobile phones and computers. They were also asked to evaluate ALICE (its performance, functionality, usability, reliability, acceptability, usefulness, design, simplicity, accessibility, and problem-solving power as well as overall satisfaction with the tool), whether they would recommend the app to relatives, friends and acquaintances, and their perception of the degree of independence they had achieved thanks to ALICE
4b-i) Report if outcomes were (self-)assessed through online questionnaires
None
4b-ii) Report how institutional affiliations are displayed
Not at all important
5) CONSORT: Describe the interventions for each group with sufficient details to allow replication, including how and when they were actually administered
5-i) Mention names, credential, affiliations of the developers, sponsors, and owners
To evaluate ALICE we opted for a single-blind experimental design with two groups (control and experimental) and pre- and- post assessments. Patients were randomly assigned to the control or experimental group. The control group was composed of subjects who did not use ALICE, and the experimental group of people used this tool for three months. To maintain the single-blind and be able to link the pre- and post- measurements, patient were assigned codes as a function of their date of birth and initials
Those in the control group received oral and written information regarding the main risks related to their medications and the most common errors of patients when taking medications. Participants in the experimental group were given a BQ tablet or an iPad with the ALICE app installed and personalised according to the medications they had been prescribed as listed in their medical record. Patients in this group attended individual sessions of up to two hours to be shown how to use the app. During the study period, patients from the experimental group had a contact telephone number for any query regarding the use of the tablet or the app
5-ii) Describe the history/development process
ALICE design
A tablet-based medication self-management app (called ALICE) was designed to help patients to remember to take all their medications, at the correct doses, distinguish between drugs to avoid confusions, avoid known potential interactions and common errors in use of the medications and know how to properly store the medications. ALICE was also designed to remember doctors' recommendations on healthy habits such as physical exercise and diet. The app design was based on suggestions extracted from 3 nominal groups with a total of 23 patients and a focus group with 7 professionals (3 physicians and 4 pharmacists). The guide with questions to be posed to patients and professionals was drawn up considering the classification of medication errors in the study by Fields at the Meyers Primary Care Institute, Massachusetts13 and previous research by our group on patients' perception of safety and the frequency and characteristics of patients' medication errors3.

Tablets used
The tablet used was selected on the basis of the need for a device with an at least 7-inch, easy to use touch screen, ensuring that users would only have to follow simple instructions and tap on some icons on the screen. Specifically, the BQ Verne Plus 3G with an LCD Tactile screen was chosen in the case of Android, and an iPad 2 with Wi-Fi + 3G in the case of the iOS.

ALICE characteristics
ALICE was designed to work with personalised prescriptions and recommendations given to patients, with a function making it possible to store details of all their prescriptions and related instructions, as well as images for each of the medications (even allowing pictures to be taken of the packaging or the appearance of the medication itself), and recommendations of the various different doctors seeing the same patient. A second function established a customized system of alerts and reminders for a given alert, to remind patients when to take their medications and to put into practice healthy habits, using the approaches suggested by participants in the nominal groups (for example, the association of the intake of medicines with particular meals or daily activities, and various techniques for splitting pills). Lastly, a third function was to enable monitoring of the level of adherence to the prescriptions and medical advice, the tablet connecting via a wireless or 3G network with the study monitoring system, with the GP and with relative or caregiver when authorized by the patient.
Once the tool had been designed and prior to the experimental phase, we assessed its feasibility, verifying that all the app characteristics proposed in the previous phase had in fact been included in the design process. For this purpose, eight elderly patients assessed the user-friendliness of the tool, its degree of intuitiveness, whether the font was sufficiently large, the contrast of the text and images, and the quality of the photographs.
ALICE had the following features: a user-friendly interface to introduce text and images; various medication reminder alerts and messages for patients, including sounds or flashing and also messages sent to relatives or caregivers; and a complete list for the caregivers of all prescriptions regardless of the number of doctors involved as well as a summary of the patient adherence behaviour.
5-iii) Revisions and updating
Once the tool had been designed and prior to the experimental phase, we assessed its feasibility, verifying that all the app characteristics proposed in the previous phase had in fact been included in the design process. For this purpose, eight elderly patients assessed the user-friendliness of the tool, its degree of intuitiveness, whether the font was sufficiently large, the contrast of the text and images, and the quality of the photographs
5-iv) Quality assurance methods

All the participating patients completed a questionnaire to assess the rates of missed doses and of medication errors, and adherence to treatment (assessed using the Morisky Medication Adherence 4 items Scale -MMAS-4-), as well as self-perceived health status, the number of doctors seen, whether they used a physical pillbox, and who organized their medication (considering the previous three months). Additionally, we recorded data on the sociodemographic characteristics of participants, namely sex, age and civil status. Those in the control group received oral and written information regarding the main risks related to their medications and the most common errors of patients when taking medications. Participants in the experimental group were given a BQ tablet or an iPad with the ALICE app installed and personalised according to the medications they had been prescribed as listed in their medical record. Patients in this group attended individual sessions of up to two hours to be shown how to use the app. During the study period, patients from the experimental group had a contact telephone number for any query regarding the use of the tablet or the app.
Three months later, the measurements made pre-intervention were repeated and extra information was collected regarding treatment adherence based on the data provided by ALICE (post). Additionally, subjects from the experimental group answered a series of questions to ascertain whether they, prior to the study, had experience of using tablets, smartphones, mobile phones and computers. They were also asked to evaluate ALICE (its performance, functionality, usability, reliability, acceptability, usefulness, design, simplicity, accessibility, and problem-solving power as well as overall satisfaction with the tool), whether they would recommend the app to relatives, friends and acquaintances, and their perception of the degree of independence they had achieved thanks to ALICE
To maintain the single-blind and be able to link the pre- and post- measurements, patient were assigned codes as a function of their date of birth and initials

5-v) Ensure replicability by publishing the source code, and/or providing screenshots/screen-capture video, and/or providing flowcharts of the algorithms used

Not at all important

5-vi) Digital preservation

Not at all important

5-vii) Access

Participants in the experimental group were given a BQ tablet or an iPad with the ALICE app installed and personalised according to the medications they had been prescribed as listed in their medical record. Patients in this group attended individual sessions of up to two hours to be shown how to use the app. During the study period, patients from the experimental group had a contact telephone number for any query regarding the use of the tablet or the app

5-viii) Mode of delivery, features/functionalities/components of the intervention and comparator, and the theoretical framework

ALICE was designed to work with personalised prescriptions and recommendations given to patients, with a function making it possible to store details of all their prescriptions and related instructions, as well as images for each of the medications (even allowing pictures to be taken of the packaging or the appearance of the medication itself), and recommendations of the various different doctors seeing the same patient. A second function established a customized system of alerts and reminders for a given alert, to remind patients when to take their medications and to put into practice healthy habits, using the approaches suggested by participants in the nominal groups (for example, the association of the intake of medicines with particular meals or daily activities, and various techniques for splitting pills). Lastly, a third function was to enable monitoring of the level of adherence to the prescriptions and medical advice, the tablet connecting via a wireless or 3G network with the study monitoring system, with the GP and with relative or caregiver when authorized by the patient.
ALICE had the following features: a user-friendly interface to introduce text and images; various medication reminder alerts and messages for patients, including sounds or flashing and also messages sent to relatives or caregivers; and a complete list for the caregivers of all prescriptions regardless of the number of doctors involved as well as a summary of the patient adherence behaviour

5-ix) Describe use parameters

Three months later, the measurements made pre-intervention were repeated and extra information was collected regarding treatment adherence based on the data provided by ALICE (post).

5-x) Clarify the level of human involvement

The app design was based on suggestions extracted from 3 nominal groups with a total of 23 patients and a focus group with 7 professionals (3 physicians and 4 pharmacists).

5-xi) Report any prompts/reminders used

During the study period, patients from the experimental group had a contact telephone number for any query regarding the use of the tablet or the app

5-xii) Describe any co-interventions (incl. training/support)

None

6a) CONSORT: Completely defined pre-specified primary and secondary outcome measures, including how and when they were assessed

In order to assess the effectiveness of ALICE, we built various univariate linear models and, where there were inter-group differences in pre-intervention measurements, performed univariate linear models and ANOVA, using as the dependent variables the differences between the pre- and post-intervention measurements in: MMAS-4scores, self-perceived health status, levels of glycosylated haemoglobin (HbA1), cholesterol (LDL), and blood pressure, number of medication errors and missed doses related to medication reported by patients. Allocation to the experimental or control group was considered as the independent variable. In the experimental group, we carried out Pearson's correlation analysis between MMAS-4scores and treatment adherence assessed objectively in terms of the number of alerts that had not been acknowledged or had been acknowledged late (data provided by ALICE). Additionally, to allow for the potential effect of the level of experience of patients with ICTs, we compared the pre- and post-intervention measurements in the experimental group adjusting for the effect of familiarity with ICTs. We compared the rating of ALICE by patients in the experimental group with and without ICT experience using the chi-square test and their level of satisfaction with ALICE (on a scale of 0 to 10) using the Student's t-test. In all cases, we checked the assumptions of the statistical tests used were met. We considered $p \leq 0.05$ to be statistically significant

6a-i) Online questionnaires: describe if they were validated for online use and apply CHERRIES items to describe how the questionnaires were designed/deployed

None online

6a-ii) Describe whether and how “use” (including intensity of use/dosage) was defined/measured/monitored

None

6a-iii) Describe whether, how, and when qualitative feedback from participants was obtained

Telephone contact was useful to know how Alice works but data was collected through interviews sung structured questionnaires.

6b) CONSORT: Any changes to trial outcomes after the trial commenced, with reasons

None

7a) CONSORT: How sample size was determined

7a-i) Describe whether and how expected attrition was taken into account when calculating the sample size

The sample size was calculated to detect a difference between means of at least of 10 points with a statistical power of 90% at a level of significance of $\alpha = 0.05$ (in a two-tailed test).

7b) CONSORT: When applicable, explanation of any interim analyses and stopping guidelines

None

8a) CONSORT: Method used to generate the random allocation sequence
randomly selection of patients
8b) CONSORT: Type of randomisation; details of any restriction (such as blocking and block size)
randomly without any restriction. We defined the following inclusion criteria for the study: multimorbid patients taking multiple medications, over 65 years old, with a Barthel score of more than 60, living in their own home and able to manage the administration of their medication at home
9) CONSORT: Mechanism used to implement the random allocation sequence (such as sequentially numbered containers), describing any steps taken to conceal the sequence until interventions were assigned
randomly of patients attending their doctor
10) CONSORT: Who generated the random allocation sequence, who enrolled participants, and who assigned participants to interventions
JJM y AP. Patients were randomly assigned to the control or experimental group.
AP was the only person that knew if patients belongs to the experimental or control groups because she was attended them. AP was a person contracted for this project who did not intervene as a researcher in any of the phases of the study
11a) CONSORT: Blinding - If done, who was blinded after assignment to interventions (for example, participants, care providers, those assessing outcomes) and how
11a-i) Specify who was blinded, and who wasn't
Patients were randomly assigned to the control or experimental group.
AP was the only person that knew if patients belongs to the experimental or control groups because she was attended them. AP was a person contracted for this project who did not intervene as a researcher in any of the phases of the study
11a-ii) Discuss e.g., whether participants knew which intervention was the "intervention of interest" and which one was the "comparator"
Yes, but experimental subject did not know the control group and control subject did not know the existence of ALICE
11b) CONSORT: If relevant, description of the similarity of interventions
Those in the control group received oral and written information regarding the main risks related to their medications and the most common errors of patients when taking medications. Participants in the experimental group were given a BQ tablet or an iPad with the ALICE app installed and personalised according to the medications they had been prescribed as listed in their medical record. Patients in this group attended individual sessions of up to two hours to be shown how to use the app
12a) CONSORT: Statistical methods used to compare groups for primary and secondary outcomes
In order to assess the effectiveness of ALICE, we built various univariate linear models and, where there were inter-group differences in pre-intervention measurements, performed univariate linear models and ANOVA, using as the dependent variables the differences between the pre- and post-intervention measurements in: MMAS-4scores, self-perceived health status, levels of glycosylated haemoglobin (HbA1), cholesterol (LDL), and blood pressure, number of medication errors and missed doses related to medication reported by patients. Allocation to the experimental or control group was considered as the independent variable. In the experimental group, we carried out Pearson's correlation analysis between MMAS-4scores and treatment adherence assessed objectively in terms of the number of alerts that had not been acknowledged or had been acknowledged late (data provided by ALICE). Additionally, to allow for the potential effect of the level of experience of patients with ICTs, we compared the pre- and post-intervention measurements in the experimental group adjusting for the effect of familiarity with ICTs.
12a-i) Imputation techniques to deal with attrition / missing values
All used ALICE during this study
12b) CONSORT: Methods for additional analyses, such as subgroup analyses and adjusted analyses
We compared the rating of ALICE by patients in the experimental group with and without ICT experience using the chi-square test and their level of satisfaction with ALICE (on a scale of 0 to 10) using the Student's t-test

RESULTS

13a) CONSORT: For each group, the numbers of participants who were randomly assigned, received intended treatment, and were analysed for the primary outcome
Data were obtained from 99 patients (48 and 51 in the control and experimental groups, respectively).
13b) CONSORT: For each group, losses and exclusions after randomisation, together with reasons
Lost to follow-up 0
13b-i) Attrition diagram
Figure 1
14a) CONSORT: Dates defining the periods of recruitment and follow-up
Each patients used ALICE three months. This study was carried out between May 2013 and March 2014 because the same procedure was applied to patients according where they have their health center
14a-i) Indicate if critical "secular events" fell into the study period
Christmas but no patient was using ALICE on Christmas
14b) CONSORT: Why the trial ended or was stopped (early)
No
15) CONSORT: A table showing baseline demographic and clinical characteristics for each group
table 1
15-i) Report demographics associated with digital divide issues
Experience with ICTs has been described, table 3
16a) CONSORT: For each group, number of participants (denominator) included in each analysis and whether the analysis was by original assigned groups
16-i) Report multiple "denominators" and provide definitions
Effects of ALICE
Patients from the experimental group reported greater treatment adherence (measured using the Morisky Medication Adherence Scale) and a lower rate of missed doses at the end of the study (Table 2). Specifically, treatment adherence (in terms of the scale scores) increased by 28.3% and the rate of missed doses fell by 27.3%. On the other hand, the univariate linear analysis of pre-post differences in the control and experimental groups indicated that ALICE was not effective in reducing the rate of medication errors
16-ii) Primary analysis should be intent-to-treat
Effects of ALICE
Patients from the experimental group reported greater treatment adherence (measured using the Morisky Medication Adherence Scale) and a lower rate of missed doses at the end of the study (Table 2). Specifically, treatment adherence (in terms of the scale scores) increased by 28.3% and the rate of missed doses fell by 27.3%. On the other hand, the univariate linear analysis of pre-post differences in the control and experimental groups indicated that ALICE was not effective in reducing the rate of medication errors
17a) CONSORT: For each primary and secondary outcome, results for each group, and the estimated effect size and its precision (such as 95% confidence interval)

table 2 and table 3
17a-i) Presentation of process outcomes such as metrics of use and intensity of use
Not at all important
17b) CONSORT: For binary outcomes, presentation of both absolute and relative effect sizes is recommended
Not applied
18) CONSORT: Results of any other analyses performed, including subgroup analyses and adjusted analyses, distinguishing pre-specified from exploratory
Tables 3, 4 and 5
18-i) Subgroup analysis of comparing only users
Table 5
19) CONSORT: All important harms or unintended effects in each group
Not applied
19-i) Include privacy breaches, technical problems
see table 5
19-ii) Include qualitative feedback from participants or observations from staff/researchers
Yes because ALICE was designed using qualitative techniques participating patients and healthcare providers

DISCUSSION

20) CONSORT: Trial limitations, addressing sources of potential bias, imprecision, multiplicity of analyses
20-i) Typical limitations in ehealth trials
Limitations
This type of app does not address the issue of where to store medication at home to have it accessible when it should be taken. The data collected do not include details of the number of drugs to be taken daily. No count was made of pills and nor did we use any other system to check that, in addition to acknowledging the alert, patients actually took any medication. Further, we cannot be sure that they took the correct dose or even drug. We do not know whether ALICE would continue to be effective in the longer term. This is important, as one notable reason patients cite for stopping taking medications is the feeling that they are not working for them. There is some evidence that the Morisky Medication Adherence Scale overestimates adherence, yielding higher rates than those obtained from pill counts. This study did not consider medication reconciliation. In some cases, if there has been inappropriate prescribing, following treatment regimens could pose a risk. This app only addresses non-intentional non-adherence. In the event of a patient being reluctant or opposed to taking a certain medicine (this being known to affect around 43% of cases, this type of tool might be useful, for its educational features, but exploring this issue is beyond the scope of this study
21) CONSORT: Generalisability (external validity, applicability) of the trial findings
21-i) Generalizability to other populations
Most apps have been designed for patients with less complex health problems and/or experience with ICTs. This study should change the expectations of developers and mobile phone companies, encouraging them to develop apps, as well as devices, suited to older patients with multimorbidity who are normally excluded from studies for being too complex, as such tools could improve the capacity of such individuals to manage their illnesses.
21-ii) Discuss if there were elements in the RCT that would be different in a routine application setting
Glucose monitors and other devices currently in use in telemedicine programmes could have add-ons to help individual patients use their medications more safely. Such devices should not only include alerts for medicines, but also reminders to put advice on healthy habits into practice
22) CONSORT: Interpretation consistent with results, balancing benefits and harms, and considering other relevant evidence
22-i) Restate study questions and summarize the answers suggested by the data, starting with primary outcomes and process outcomes (use)
Treatment adherence was getter in patients in the experimental group than controls. It is important to highlight that ALICE helps to solve a common problem in elderly patients, namely, remembering whether they have taken their medication. Using ALICE, the number of errors decreases, although the patients who benefited in this respect were those who previously made the most errors. Our data suggest that ALICE does contribute to reducing systematic errors but not all medication errors
22-ii) Highlight unanswered new questions, suggest future research
Further studies on virtual pillboxes for tablets and smartphones could explore whether adherence can be improved by personalization of treatment regimens as suggested by other authors . Specifically, future research should assess to what extent these tools are useful for older individuals living alone, a situation that is expected to be the reality for a growing number of patients in the near future

Other information

23) CONSORT: Registration number and name of trial registry
This study was funded by the Spanish Health Ministry, reference EC11-527. This is a serie of controlled trials without any other sources of funding to test new ideas, products, etc
24) CONSORT: Where the full trial protocol can be accessed, if available
The trial protocol is available upon request. It's in Spanish but we can translate.
25) CONSORT: Sources of funding and other support (such as supply of drugs), role of funders
This study was funded by the Spanish Health Ministry, reference EC11-527
X26-i) Comment on ethics committee approval
The procedure was approved by the Ethics Committee of Miguel Hernández University (DPS-JJM-003-11).
x26-ii) Outline informed consent procedures
We requested the informed consent of patients from both control and experimental groups.
X26-iii) Safety and security procedures
During the study period, patients from the experimental group had a contact telephone number for any query regarding the use of the tablet or the app
X27-i) State the relation of the study team towards the system being evaluated
None
